# *Cis*-encoded non-coding antisense RNAs in streptococci and other low GC Gram (+) bacterial pathogens

**DOI:** 10.3389/fgene.2015.00110

**Published:** 2015-03-26

**Authors:** Kyu Hong Cho, Jeong-Ho Kim

**Affiliations:** ^1^Department of Biology, Indiana State UniversityTerre Haute, IN, USA; ^2^Department of Biochemistry and Molecular Medicine, The George Washington University School of Medicine and Health ScienceWashington, DC, USA

**Keywords:** non-coding RNAs, regulatory RNAs, antisense RNAs, streptococci, Gram (+) pathogens

## Abstract

Due to recent advances of bioinformatics and high throughput sequencing technology, discovery of regulatory non-coding RNAs in bacteria has been increased to a great extent. Based on this bandwagon, many studies searching for *trans*-acting small non-coding RNAs in streptococci have been performed intensively, especially in the important human pathogen, group A and B streptococci. However, studies for *cis*-encoded non-coding antisense RNAs in streptococci have been scarce. A recent study shows antisense RNAs are involved in virulence gene regulation in group B streptococcus, *S. agalactiae*. This suggests antisense RNAs could have important roles in the pathogenesis of streptococcal pathogens. In this review, we describe recent discoveries of chromosomal *cis*-encoded antisense RNAs in streptococcal pathogens and other low GC Gram (+) bacteria to provide a guide for future studies.

## Introduction

Non-coding regulatory RNAs exist in all three kingdoms and confer another layer of regulation mechanism for gene expression. Generally, the regulation by non-coding RNAs occurs at a post-transcriptional level, so their regulation would be fast and effective. Bacteria produce three general groups of non-coding regulatory RNAs: (i) *cis*-acting 5′ element non-coding RNAs, (ii) *trans*-acting small non-coding RNAs, and (iii) *cis*-encoded antisense RNAs. A c*is*-acting 5′ non-coding RNA is usually attached to the 5′ side of an mRNA whose expression is regulated by the non-coding RNA. A structural change of the non-coding RNA occurs by binding to small metabolites (riboswitches), or by change of temperature (thermoregulators) or pH (pH sensors). The structural change influences transcription or translation of the downstream gene or genes in an operon. *Trans*-acting small non-coding RNAs are usually encoded in intergenic regions on the chromosome and control translation or degradation of their target mRNAs. Generally, each *trans*-acting non-coding RNA has multiple target mRNAs and binds near the ribosomal binding site of the target mRNAs. A c*is*-acting antisense RNA (antisense RNA) is expressed as a complementary sequence of an mRNA that becomes the sole target RNA.

Previously, these non-coding RNAs had been discovered by computational predictions coupled with expression studies, microarrays, sequencing of small sized cDNA libraries, and high throughput sequencing approaches. Due to recent technological advances of tiling microarray, RNA deep sequencing, and bioinformatics, the search for non-coding regulatory RNAs on a genome-wide scale has been actively performed. As a result, the functions and regulatory mechanisms of discovered non-coding regulatory RNAs are widely studied. However, because of technical difficulties to distinguish the source of expressed RNAs between the two DNA strands, the search for antisense RNAs using high throughput methods has been retarded, compared to the search for *trans*-acting small RNAs. This makes antisense RNAs the least studied non-coding RNAs in streptococci to date. Currently no systematic search for antisense RNAs has been done in *S. pyogenes*, and only one search has been performed in *S. agalactiae*.

Considerable antisense transcription has been discovered in both eukaryotes and prokaryotes. The number of *cis*-encoded antisense RNAs in bacteria was once considered much smaller than that of eukaryotes due to the compact organization of protein-coding genes in the chromosome. However, recent studies indicate bacteria also produce a number of *cis*-encoded antisense RNAs. Bacterial *cis*-encoded antisense RNAs were discovered several decades ago, and most antisense RNAs were expressed from mobile genetic elements such as plasmids, phages, and transposons (Brantl, 2007). Since antisense RNAs expressed from bacterial chromosomes had not been discovered, it was thought that antisense RNAs were not generally used to control chromosomal gene expression in bacteria. However, during recent decades, many RNAs antisense to chromosomal genes have been discovered in bacteria. The other kingdom of prokaryotic microorganisms, archaea, also express *cis*-encoded antisense transcripts. An archaeal organism, *Sulfolobus solfataricus* P2, expresses about 310 non-coding RNAs and among these non-coding RNAs, almost 60% (185 non-coding RNAs) are *cis*-encoded antisense RNAs (Wurtzel et al., 2010). Although many antisense RNAs have been discovered in prokaryotes recently, their functions and regulation mechanisms are largely not studied.

**Figure 1:**
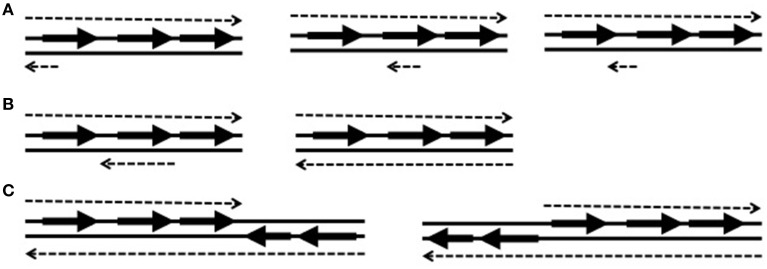
**Examples of antisense RNA types illustrated with a three-gene operon**. The solid lines depict double stranded DNA with genes (arrows). Each dotted line represents expressed RNA matching with the sequence of each DNA strand. The top dotted lines are mRNAs and the bottom dotted lines are *cis*-encoded antisense RNAs. **(A)** Small antisense RNAs complementary to the sequence of ribosome-binding site (RBS), in the middle of a gene, or of an intergenic region. **(B)** Long antisense RNAs complementary to an entire gene or an operon. **(C)** Excludons containing genes at its 5′ or 3′ side.

Most *cis*-encoded antisense RNAs are complementary to a small portion of an open reading frame (ORF) and often the complementary portion includes the ribosome-binding site (Figure 1A). These small antisense RNAs are widely expressed on the chromosomes, plasmids, and transposons. However, some antisense RNAs are longer than typical ones and even reach several kilobases. Long antisense RNAs can be complementary to an entire gene or genes (Figure 1B). Among long antisense RNAs, some contains the sequence of a neighboring ORF or ORFs on their 5′ or 3′ side (Figure 1C). These type of antisense RNAs, which were named excludons (Sesto et al., 2013), have been discovered only on the chromosomes of several bacteria such as *Listeria monocytogenes* (Toledo-Arana et al., 2009), *Bacillus subtilis* (Rasmussen et al., 2009), a cyanobacterium *Synechocystis sp*. (Stazic et al., 2011), and *Staphylococcus aureus* (Beaume et al., 2010). However, as more bacteria are searched for antisense RNAs, more excludons are expected to be discovered.

## Cis-encoded antisense RNAs in streptococci and other low GC gram (+) bacteria

*S. agalactiae* (Group B Streptococcus, GBS), which is an opportunistic pathogen and causative agent of bacterial sepsis, pneumonia, and meningitis in newborns, employs antisense RNAs to control virulence factors (Pichon et al., 2012). In the study of Pichon et al. they used an *in silico* method to find small non-coding RNAs and predicted the existence of 63 antisense RNAs (Table 1). They validated the existence of these antisense RNAs by verifying three of them through northern blotting (Table 2). The three RNAs, which have the sizes of 123 bps, 239 bps, and 243 bps, are fully or partially antisense to coding sequences (CDSs) involved in the pathogenicity of *S. agalactiae*. When they overexpressed two of these antisense RNAs using a multi-copy plasmid, one reduced the expression of the adjacent target gene but the other increased the expression of its target gene. This shows that antisense RNAs can carry out both negative and positive regulation.

**Table 1:** **High throughput searches for chromosomal *cis*-encoded antisense RNAs in low GC Gram-positive bacteria**.

**Bacterium**	**Total number of antisense RNAs discovered or predicted**	**Search method [references]**
*Bacillus subtilis*	143	High density tiling microarray covering both strands (Rasmussen et al., 2009) Differential RNA-seq (Irnov et al., 2010)
*Listeria monocytogenes*	10	Tiling microarray covering both strands (Toledo-Arana et al., 2009)
*Staphylococcus aureus*	113	Sequencing cDNA libraries and northern blotting (Abu-Qatouseh et al., 2010) Illuminar RNA-seq with orientation protocol (Beaume et al., 2010)
*Streptococcus agalactiae*	63	*In silico* prediction (Pichon et al., 2012)

**Table 2:** **Chromosomal *cis*-encoded antisense RNAs in low GC Gram-positive bacteria**.

**Bacterium**	**Name of antisense RNA**	**Gene (protein) antisense to**	**Size (bases)**	Discovered method^*^	**Validation method**	**References**
*Bacillus subtilis*	ncr2706	*ywqA*	47	RNA-seq		Irnov et al., 2010
	ncr1430	*bglP*	70			
	ncr1687	*wprA*	24			
	ncr1265	*yutK*	218			
	ncr2153	*comER*	101			
	ncr1186	*nadB*	17			
	ncr1006	*yoeA*	219			
	ncr1799	*mutS*	25			
	ncr2058	*yqzJ*	110			
	ncr2160	*sda*	259			
	ncr1351	*mbl*	227			
	ncr1565	*yddR*	61			
	ncr2885	*yyaQ*	106			
	ncr1546	*mtlD*	50			
	ncr507	*yfhD*	30			
	ncr2410	*ytoA*	249			
*Bacillus subtilis*	shd1	yaaC	681	Tiling microarray		Rasmussen et al., 2009
	shd2	*dck*	681			
	shd3	*yabD yabE*	813			
	shd4	*yabE*	1121			
	shd5	*coaX hslO yacD*	2816			
	shd6	*lysS*	681			
	shd7	*ybaC*	1187			
	shd8	*ybbB*	461			
	shd9	*ybfG ybfH*	3233			
	shd10	*nagBB*	1077			
	shd11	*ycbR*	263			
	shd12	*yceJ*	1319			
	shd13	*nasE nasD*	813			
	shd14	*yckC yckD bglC*	2675			
	shd15	*tlpC*	1759			
	shd16	*hxlB hxlA*	1452			
	shd17	*hxlR*	417			
	shd18	*ycxD*	461			
	shd19	*yczM yczN*	439			
	shd20	*kipR lipC*	836			
	shd21	*ydbM*	791			
	shd22	*ydbO*	527			
	shd23	*ndoA rsbRA*	1099			
	shd24	*ydcO*	241			
	shd25	*vmlR*	637			
	shd26	*ydiF*	285			
	shd27	*ydzW ydzW ydzW ydzW*	527			
	shd28	*ydjE*	373			
	shd29	*yebD yebE yebG*	1077			
	shd30	*yerA*	351			
	shd31	*yeeD yezA*	791			
	shd32	*yeeK*	263			
	shd33	*lplD yetF*	1583			
	shd34	*yfmG*	461			
	shd35	*yfhK yfhL yfhM*	1583			
	shd36	*ygaB*	417			
	shd37	*ygaJ*	636			
	shd38	*ygaK*	967			
	shd39	*nhaC*	197			
	shd40	*yhfA*	1495			
	shd41	*yisI*	483			
	shd42	*yisL*	593			
	shd43	*yisQ*	769			
	shd44	*yitZ*	703			
	shd45	*yjzC*	329			
	shd46	*yjaZ*	857			
	shd47	*yjbB*	1209			
	shd48	*yjbE*	835			
	shd49	*yjcK yjcL*	1915			
	shd50	*ykuT*	923			
	shd51	*ylaK*	307			
	shd52	*ctaA*	681			
	shd53	*yloB*	659			
	shd54	*ymfJ*	373			
	shd55	*yncF*	593			
	shd56	*yneE*	615			
	shd57	*cotM sspP sspO*	879			
	shd58	*yogA*	615			
	shd59	*yoaE yoaF*	1252			
	shd60	*yoqZ yoqY*	637			
	shd61	*yonT*	417			
	shd62	*blyA bhlA bhlB*	1517			
	shd63	*yokD*	549			
	shd64	*dinF*	1187			
	shd65	*yppC*	373			
	shd66	*ponA*	351			
	shd67	*birA*	197			
	shd68	*yqxK*	483			
	shd69	*yqjF*	901			
	shd70	*yqjD*	373			
	shd71	*yqjB yqjA*	1504			
	shd72	*yqiG*	696			
	shd73	*yqhR*	725			
	shd74	*yqzG*	241			
	shd75	*yqhB*	637			
	shd76	*yqgE*	1451			
	shd77	*sigA*	967			
	shd78	*dgkA*	241			
	shd79	*comEC*	527			
	shd80	*yqdB*	219			
	shd81	*ncr58/bsrH*	549			
	shd82	*yrrI*	483			
	shd83	*leuA ilvC*	1693			
	shd84	*ytoI*	725			
	shd85	*ytrP*	637			
	shd86	*ytoP*	461			
	shd87	*ytlD*	769			
	shd88	*ythA ythB ytzL*	1715			
	shd89	*yugH*	1055			
	shd90	*yufK*	659			
	shd91	*mrpE mrpF mrpG*	901			
	shd92	*yueB*	1847			
	shd93	*yukB*	769			
	shd94	*yutK*	681			
	shd95	*yuzB*	593			
	shd96	*yutH*	527			
	shd97	*yurQ yurR*	1033			
	shd98	*yuzK yurZ metN*	1099			
	shd99	*yusW*	615			
	shd100	*cssS*	769			
	shd101	*nhaK*	571			
	shd102	*opuBD opuBC opuBB*	1957			
	shd103	*yvaV*	373			
	shd104	*sdpI sdpR*	842			
	shd105	*araE*	879			
	shd106	*yvfU*	285			
	shd107	*cwlO*	395			
	shd108	*yvjA prfB*	1209			
	shd109	*comFC comFB comFA yviA*	3516			
	shd110	*tuaH*	373			
	shd111	*tuaA*	329			
	shd112	*ggaA*	1319			
	shd113	*spo0F*	593			
	shd114	*narK*	461			
	shd115	*ywfM ywfL cysL*	2903			
	shd116	*pta*	505			
	shd117	*bacF*	593			
	shd118	*yxlH*	681			
	shd119	*cimH yxkI yxzE*	2661			
	shd120	*yxkA*	725			
	shd121	*yxjA*	637			
	shd122	*yxxF*	1055			
	shd123	*yxeA yxdM yxdL*	2309			
	shd124	*yybT yybS*	1429			
	shd125	*yybI*	615			
	shd126	*yyaM*	461			
	shd127	*jag*	549			
*Listeria monocytogenes*	SRP	Partially antisense to *lmo2711*	332	Tiling microarray		Toledo-Arana et al., 2009
	rli23	*lmo0172* (Transposase)	97			
	rli25	*lmo0330* (Transposase)	102			
	rli29	Antisense to the 5′UTR of *lmo0471*	193			
	rli30	*lmo0506*	115			
	rli35	*lmo0828* (Transposase)	102			
	rli45	Antisense to rli46 (small non-coding RNA)	77			
	rli46	Antisense to rli45	294			
	Anti2095-8 RNA1 RNA2	*lmo2095 lmo2095-8*	255 2149			
	Anti2325-7 RNA1 RNA2	*lmo2325 lmo2325-7*	264 995			
	Anti2394-5 RNA1 RNA2	*lmo2394 lmo2394-5*	216 693			
*Staphylococcus aureus*	Sau-13	*SA2421*	110; 140; 210	cDNA library Sequencing	Northern blot	Abu-Qatouseh et al., 2010
	Sau-31	*SA2021*	210			
	Sau-50	*hu* (DNA-binding prtein II*)*	210			
	Sau-53	*argC*	200			
	Sau-59	*SA0931*	130			
	Sau-66	*SA0671*	210			
*Staphylococcus aureus*	Teg5as	*SA0024*	330	RNA-seq		Beaume et al., 2010
	Teg6as	*SA0025*	405			
	Teg7as	*SA0027* and *SA0026*	36			
	Teg8as	*SAS002* and *SA0028*	84			
	Teg10as	*SA0044*	42			
	Teg14as	*SA0062*	143			
	Teg15as	*SA0097* and *SA0098*	72			
	Teg16as	*SA0101* and *SA0100*	81			
	Teg17as	*capM*	108			
	Teg18as	*SA0306*	864			
	Teg19as	*SA0412* and *SA0413*	2475			
	Teg20as	*SA0620*	1008			
	Teg21as	*SA1825*	63			
	Teg22as	*SA1830*	63			
	Teg23as	*nrgA*	36			
	Teg25as	*SA2200*	117			
	Teg26as	*SA2218*	63			
	Teg27as	*SA2224*	90			
	Teg28as	*SA2440*	36			
	Teg36as	*ssaA*	448			
	Teg37as	*SA0970*	108			
	Teg38as	*SA0351*	50			
	Teg10aspl	*SAP031*	36			
	Teg39as	*SA0031*	210			
	Teg40as	*SA0751*	299			
	Teg41as	*SAS024*	141			
*Streptococcus agalactiae*	SQ18	*gbs0031* (Surface exposed protein	123	*In Silico* prediction	Northern blot	Pichon et al., 2012
	SQ407	*lmb* (Laminin binding protein)	239			
	SQ485	*gbs1558*/1559 (putative ABC transporter)	242			
*Streptococcus mutans*	srSm	Fst-Sm (Fst-like toxin)	70	PSI-BLAST and TBLAST	Northern blot	Koyanagi and Levesque, 2013

* Putative antisense RNAs predicted by in silico or cDNA library sequencing without any validation are not listed in this table.

On the other hand, the discovery of antisense RNAs in another important streptococcal pathogen *Streptococcus pyogenes* (Group A Streptococcus, GAS) has not been reported. Many studies have been done to search for *trans-*acting small non-coding RNAs, but no systematic study has been done so far to search for antisense RNAs. Thus, it is not known if antisense RNAs in this pathogen have an important role in controlling gene expression and/or virulence.

An RNA-based toxin-antitoxin system was discovered on the chromosome of *Streptococcus mutans*, an oral streptococcal pathogen (Table 2) (Koyanagi and Levesque, 2013). This is an unusual case because most toxin-antitoxin systems in bacteria are encoded in plasmids. The *S. mutans* antitoxin is an antisense RNA (srSm) converging toward the end of the gene of Fst-like toxin (Fst-Sm), so the expression of the antitoxin antisense RNA inhibits the production of Fst-like toxin.

High throughput searches for non-coding regulatory RNAs in *Bacillus subtilis* have been performed to gain more knowledge on the regulation of gene expression by non-coding RNAs in this low GC Gram (+) model organism (Rasmussen et al., 2009; Irnov et al., 2010). In these searches, Rasmussen et al. discovered 127 antisense RNAs through a high density tiling array (Rasmussen et al., 2009), and then Irnov et al. discovered 16 novel antisense RNAs using a differential RNA-seq analysis (Table 1) (Irnov et al., 2010). The results from these studies reveal that target genes of antisense RNAs are involved in stress response, sporulation, and expression of SigA, the principal sigma factor during vegetative growth (Table 2). Therefore, antisense RNAs in *B. subtilis* appear to influence a variety of important regulations to adapt diverse environmental conditions.

*Staphylococcus aureus* is a remarkable opportunistic pathogen causing a broad spectrum of diseases like *S. pyogenes*, which range from superficial skin diseases to fatal systemic infections including sepsis, pneumonia, and bone infections. Since the emergence and spread of drug-resistant and community-acquired strains, *S. aureus* infections have drawn great attention. The most intensively studied non-coding RNA in *S. aureus* is RNAIII that is a regulatory RNA controlling many virulence factors as the effector of the *agr* quorum sensing system. Even though RNAIII controls translation and degradation of target mRNAs with an antisense mechanism, its action is *trans*, not *cis*, thus RNAIII is not discussed here because of the narrow scope of this review (for a review on RNAIII, see Novick and Geisinger, 2008).

Previously, several studies have been performed to discover non-coding regulatory RNAs in *S. aureus* through computational methods, sequencing of small sized cDNAs, and high throughput strand-specific RNA sequencing technology (Table 1) (Pichon and Felden, 2005; Geissmann et al., 2009; Abu-Qatouseh et al., 2010; Beaume et al., 2010; Bohn et al., 2010). From these studies, about 100 *cis*-encoded antisense RNAs have been discovered, some of which were experimentally detected by northern blotting, Rapid Amplification of cDNA Ends (RACE) mapping, or reverse transcriptase quantitative PCR (RT-qPCR) (Table 2). Many of these antisense RNAs are expressed from pathogenicity islands and mobile elements such as plasmids and transposons. Interestingly, existence of some antisense RNAs was unique in a strain, suggesting that gene regulation by *cis*-encoded antisense RNA could be strain specific. Long antisense RNAs are also present in *S. aureus*. The antisense RNA complementary to the gene encoding a secretory antigen (SA0620) is bigger than 1 kb (Beaume et al., 2010).

In the study by Beaume et al., 10 *cis*-encoded antisense RNAs out of total discovered 35 were expressed in pathogenicity islands or in the chromosome *mec* cassette, which is a mobile genetic element conferring methicillin resistance (Beaume et al., 2010). This indicates that antisense RNAs could play a key role in *S. aureus* infections. These antisense RNAs are particularly abundant in genes involved in cell wall and cell envelope biogenesis and in replication, recombination, and repair. Interestingly, two of these antisense RNAs are complementary to the small non-coding RNAs, SprA1, and AprG. These two antisense RNA-small non-coding RNA pairs are predicted to form type I toxin-antitoxin modules. The study of *S. aureus* small colony variants identified 78 antisense RNA candidates (Abu-Qatouseh et al., 2010). Some antisense RNAs in *S. aureus* are involved in the differential expression of genes in the same operon. An example is antisense RNAs complementary to a part of each *capF* and *capM* transcript of the same capsular polysaccharide synthesis operon (cap operon) (Abu-Qatouseh et al., 2010; Beaume et al., 2010). Even though they are expressed as one mRNA, the two genes are differentially translated by the antisense RNAs.

*Listeria monocytogenes* is a Gram (+) pathogenic bacterium causing food-borne infection, listeriosis, which can lead to meningitis in newborns. This pathogen has a well-defined virulence mechanism to inhibit phagolysosome formation and proliferate inside host cells, so has been extensively used as a model organism for the study of pathogen-host interaction (Hamon et al., 2006). Previously, the Cossart group examined the transcription profile of this pathogen using tiling microarrays that covered both strands of the chromosome, and discovered many non-coding RNAs including 10 *cis*-encoded antisense RNAs (Table 1). Three of them were already classified as small RNAs and seven were newly discovered (Toledo-Arana et al., 2009). Most *cis*-encoded antisense RNAs cover a small portion of an open reading frame (ORF), but three antisense RNAs are large enough to cover more than one ORF. Interestingly, all of these long antisense RNAs are expressed with a shorter antisense RNA. Both shorter and longer antisense RNAs are expressed at the same start site but they have different termination sites. The importance of these two different size antisense transcripts has not been determined yet.

## Regulation mechanisms by Cis-encoded antisense RNAs

Antisense RNAs can control gene expression by binding to their cognate sense RNAs. The binding occurs at the 5′ end, 3′ end, or in the middle of mRNAs depending on the location they are expressed (Figure 1A). Also, long antisense RNAs can overlap an entire mRNA encoding a protein or proteins (Figure 1B). The different binding locations confer different control mechanisms. Based on their binding locations on sense RNAs, antisense RNAs may act in three ways: (i) transcription terminators in the mechanism of transcription attenuation or transcription interference, (ii) potential inhibitors of translation initiation, or (iii) modulators of mRNA degradation. Antisense RNAs influence gene expression at the transcriptional or post-transcriptional level. Transcription interference and transcription attenuation occur at the transcriptional levels, and translation inhibition and mRNA degradation occur at post-transcriptional levels. The degree of control by antisense RNAs can be achieved by their differential expression level at different conditions. The expression ratio between a sense RNA and the antisense RNA will influence the expression of the sense gene.

In transcription interference, two promoters of an antisense RNA and its target sense RNA present very close in *cis*-position and their transcriptions occur in the convergent direction, and then the transcription rate from one promoter becomes suppressed by the other promoter (Callen et al., 2004). In this case, the transcription of the weaker promoter seems suppressed more. Another regulation mechanism at the transcriptional level by antisense RNAs is transcription attenuation. In transcription attenuation, an antisense RNA binds to the region in front of the Shine-Dalgano sequence of the target mRNA, and this binding induces the formation of transcription terminator structure. Hence, when the antisense RNA binds near or at the 5′ end of the cognate sense RNA, the transcription of the sense RNA is terminated (Brantl, 2002; Stork et al., 2007). In this regulation, if an antisense RNA binds an intergenic region in a polycistronic mRNA, then it can create differential gene expression between the genes located upstream and downstream of the intergenic region, and the upstream gene is more expressed than the downstream gene (Stork et al., 2007).

A common post-transcriptional level regulation by antisense RNAs is modulating translation resulting in translation inhibition or activation. In translation inhibition, antisense RNAs bind directly to the Shine-Dalgano sequence (SD sequence) of mRNAs, and inhibit ribosome-binding (Greenfield et al., 2001; Hernandez et al., 2006; Kawano et al., 2007). This inhibition of translation might increase or decrease the degradation of mRNAs by ribonuclease. In translation activation, an antisense RNA bind near the SD sequence whose access by ribosomes are blocked by a preformed stem and loop structure, then the binding of the antisense RNA frees the SD sequence (Asano et al., 1998).

As mentioned, mRNA degradation can be influenced by a bound antisense RNA. The pairs of antisense RNA–target mRNA can be substrates of RNase III, which is a double strand specific endoribonuclease. RNase III is conserved in all the three kingdoms. A previous study of *S. aureus* showed that the deletion of RNase III increased the amount of antisense transcripts, indicating that target mRNAs bound by antisense RNAs are degraded by RNase III *in vivo* (Lasa et al., 2011). Deep sequencing analysis in the same study showed that RNase III generates 22 nt long RNA fragments with 2 nucleotide 3′ overhang from the pairs of sense-antisense transcripts. Surprisingly, 75% of mRNAs are processed by RNase III, implying that antisense regulation occurs more extensively than previously thought. Studies on other bacteria also indicate that antisense transcription occurs extensively throughout the chromosome (For a review, see Georg and Hess, 2011).

Another RNase shown to be involved in degradation of sense-antisense RNA pairs is RNase E, an endoribonuclease degrading 5′ monophosphorylated mRNAs. RNase E degrades *mgtC* mRNA in *Salmonella enterica* with an unknown mechanism when the sense RNA is bound by the antisense RNA, AmgR (Lee and Groisman, 2010). RNase E is a member of the RNA degradosome in Gram (−) bacteria, a multicomponent complex that also includes an RNA helicase, RhlB, a glycolytic enzyme, enolase, and the exoribonuclease polynucleotide phosphorylase (PNPase) (Carpousis, 2007). The main function of the RNA degradosome is known to control mRNA turnover. Most Gram (+) bacteria including streptococci, bacilli, and staphylococci do not possess an RNase E homolog. However, these bacteria possess the RNA degradosome. The Gram (+) RNA degradosome contains similar kinds of components but more members, compared to the Gram (−) counterpart: four ribonuclases, RNase Y, RNase J1, J2, and PNPase; an RNA helicase, CshA; two glycolytic enzymes, phosphofructokinase (PfkA) and enolase (Lehnik-Habrink et al., 2012). RNase E is a membrane bound protein providing the major structural scaffold interacting with other components in the Gram (−) degradosome. The structure of Gram (+) RNA degradosome has not been resolved, but protein interaction studies revealed that the endoribonuclease RNase Y, a membrane anchored protein, interacts with most other components in the degradosome, so RNase Y might be the functional homolog of RNase E (Kang et al., 2010). No study has been done yet if RNase Y is also involved in the degradation of some sense-antisense RNA pairs in Gram (+) bacteria.

In Gram (−) bacteria, most small non-coding regulatory RNAs work with the RNA chaperone protein Hfq. Generally, the presence of the Hfq protein increases the stability of small non-coding RNAs and facilitates the interaction to their target mRNAs (Gottesman and Storz, 2011). However, the role of Hfq does not seem critical in Gram (+) bacteria. The role of Hfq is dispensable in *S. aureus* (Bohn et al., 2007). There have not been many studies of Hfq in terms of *cis*-encoded antisense RNAs so far, but previous studies show that some antisense RNAs interact with Hfq (Sittka et al., 2008; Lorenz et al., 2010), and Hfq is required for the function of a *cis*-encoded antisense RNA (Ross et al., 2010). Streptococci and lactobacilli do not possess any Hfq homologs, and it has not been studied if some other protein or proteins replace the role of Hfq in *trans*-acting small RNA- or *cis*-acting antisense RNA-mediated regulation. It has been suggested that the role of Hfq might be dispensable in low GC Gram (+) bacteria because non-coding RNAs in these bacteria are longer than higher GC Gram (−) bacteria to compensate for the low GC content of the pairings (Jousselin et al., 2009).

One advantage of regulation by antisense RNAs is to confer an additional layer of gene regulation like other non-coding regulatory RNAs. In concert with protein regulators, antisense RNAs can provide more precise regulation or regulation responding to different signals. Compared to *trans*-acting small non-coding RNAs, the regulation by antisense RNAs are generally more specific. Usually *trans*-acting non-coding small RNAs have multiple target mRNAs with imperfect base-pairs, but antisense RNAs usually have just one target mRNA with the complete complementary sequence. Even though we cannot completely rule out the possibility that some antisense RNAs have several targets with partial base matches by acting in *trans*, multiple targets of an antisense RNA have not been discovered yet. Another advantage of regulation by *cis*-encoded antisense RNAs is regulation speed. Like other non-coding regulatory RNAs, most antisense RNAs act at the post-transcriptional level, so the result of the action by antisense RNAs would be faster than protein transcriptional regulators.

## Perspectives

Compared to small non-coding *trans*-acting RNAs, bacterial *cis*-encoded antisense RNAs had not been studied in the genome-wide scale because of technical difficulties. However, due to the recent development of strand specific RNA sequencing and tiling microarrays covering both strands, *cis*-encoded antisense RNAs have been subjected under the genome-wide search in many bacteria. Already hundreds of bacterial antisense RNAs have been discovered and changed the concept of regulation by antisense RNAs. So far few streptococcal antisense RNAs have been discovered, but further genome-wide search would definitely find a number of antisense RNAs in this group of bacteria and promote studies to investigate the function and molecular mechanism of regulation by antisense RNAs.

### Conflict of interest statement

The authors declare that the research was conducted in the absence of any commercial or financial relationships that could be construed as a potential conflict of interest.
